# Shared and Distinct Patterns of Functional Connectivity to Emotional Faces in Autism Spectrum Disorder and Attention-Deficit/Hyperactivity Disorder Children

**DOI:** 10.3389/fpsyg.2022.826527

**Published:** 2022-03-09

**Authors:** Kristina Safar, Marlee M. Vandewouw, Elizabeth W. Pang, Kathrina de Villa, Jennifer Crosbie, Russell Schachar, Alana Iaboni, Stelios Georgiades, Robert Nicolson, Elizabeth Kelley, Muhammed Ayub, Jason P. Lerch, Evdokia Anagnostou, Margot J. Taylor

**Affiliations:** ^1^Department of Diagnostic Imaging, Hospital for Sick Children, Toronto, ON, Canada; ^2^Program in Neurosciences & Mental Health, Hospital for Sick Children, Toronto, ON, Canada; ^3^Autism Research Centre, Bloorview Research Institute, Holland Bloorview Kids Rehabilitation Hospital, Toronto, ON, Canada; ^4^Institute of Biomedical Engineering, University of Toronto, Toronto, ON, Canada; ^5^Division of Neurology, The Hospital for Sick Children, Toronto, ON, Canada; ^6^Department of Psychiatry, University of Toronto, Toronto, ON, Canada; ^7^Department of Psychiatry, The Hospital for Sick Children, Toronto, ON, Canada; ^8^Department of Psychiatry and Behavioural Neurosciences, McMaster University, Hamilton, ON, Canada; ^9^Department of Psychiatry, Western University, London, ON, Canada; ^10^Department of Psychology and Centre for Neuroscience Studies, Queen’s University, Kingston, ON, Canada; ^11^Department of Psychiatry,Queen’s University, Kingston, ON, Canada; ^12^Wellcome Centre for Integrative Neuroimaging, FMRIB, Nuffield Department of Clinical Neurosciences, University of Oxford, Oxford, United Kingdom; ^13^Mouse Imaging Centre, The Hospital for Sick Children, Toronto, ON, Canada; ^14^Department of Paediatrics, University of Toronto, Toronto, ON, Canada; ^15^Department of Medical Imaging, University of Toronto, Toronto, ON, Canada; ^16^Department of Psychology, University of Toronto, Toronto, ON, Canada

**Keywords:** ASD, ADHD, emotional face processing, functional connectivity, children, magnetoencephalography

## Abstract

Impairments in emotional face processing are demonstrated by individuals with neurodevelopmental disorders (NDDs), including autism spectrum disorder (ASD) and attention-deficit/hyperactivity disorder (ADHD), which is associated with altered emotion processing networks. Despite accumulating evidence of high rates of diagnostic overlap and shared symptoms between ASD and ADHD, functional connectivity underpinning emotion processing across these two neurodevelopmental disorders, compared to typical developing peers, has rarely been examined. The current study used magnetoencephalography to investigate whole-brain functional connectivity during the presentation of happy and angry faces in 258 children (5–19 years), including ASD, ADHD and typically developing (TD) groups to determine possible differences in emotion processing. Data-driven clustering was also applied to determine whether the patterns of connectivity differed among diagnostic groups. We found reduced functional connectivity in the beta band in ASD compared to TD, and a further reduction in the ADHD group compared to the ASD and the TD groups, across emotions. A group-by-emotion interaction in the gamma frequency band was also observed. Greater connectivity to happy compared to angry faces was found in the ADHD and TD groups, while the opposite pattern was seen in ASD. Data-driven subgrouping identified two distinct subgroups: NDD-dominant and TD-dominant; these subgroups demonstrated emotion- and frequency-specific differences in connectivity. Atypicalities in specific brain networks were strongly correlated with the severity of diagnosis-specific symptoms. Functional connectivity strength in the beta network was negatively correlated with difficulties in attention; in the gamma network, functional connectivity strength to happy faces was positively correlated with adaptive behavioural functioning, but in contrast, negatively correlated to angry faces. Our findings establish atypical frequency- and emotion-specific patterns of functional connectivity between NDD and TD children. Data-driven clustering further highlights a high degree of comorbidity and symptom overlap between the ASD and ADHD children.

## Introduction

Facial expressions facilitate social communication by providing extensive social cues offering insight into others’ emotional state and intentions; thus, emotion recognition is essential for successful social functioning (Adolphs, 2002). Impairments in face and emotional face processing are demonstrated by individuals with neurodevelopmental disorders (NDDs), including autism spectrum disorder (ASD), for whom these deficits are a central feature (Harms et al., 2010; American Psychiatric Association, 2013; Uljarevic and Hamilton, 2013), and attention-deficit/hyperactivity disorder (ADHD; Dickstein and Castellanos, 2011). Accumulating evidence suggests that these NDDs can be comorbid, and high rates of diagnostic overlap and shared symptoms are evident, likely due to shared biological mechanisms (Ronald et al., 2008; Kushki et al., 2019; Lai et al., 2019). Therefore, a more recent focus of research has investigated the shared and distinct brain features underpinning social-cognitive skills in these NDDs (Demurie et al., 2011; Tye et al., 2013; Hutchins et al., 2016; Groom et al., 2017; Vandewouw et al., 2020, 2021).

Historically, considerable research has focused on comparisons between individuals with and without ASD. Atypical activation of core and extended face processing areas, including the primary visual cortex, fusiform gyri (FG), superior temporal sulcus (STS), amygdalae and insulae in ASD compared to typical development (TD), is well established (Critchley et al., 2000a, 2000b; Haxby et al., 2000; Pierce et al., 2001; Hubl et al., 2003; Ashwin et al., 2007; Deeley et al., 2007; Gobbini et al., 2007; Pelphrey et al., 2007; Corbett et al., 2009; di Martino et al., 2009; Leung et al., 2015, 2018, 2019), with several of these studies reporting under-activation of these areas to emotional faces in children and adults with the disorder. For example, functional resonance imaging (fMRI) studies have shown that children with ASD demonstrate under-activation of the FG and the amygdalae to faces and emotional faces (Wang et al., 2004; Pierce and Redcay, 2008; Corbett et al., 2009). Corbett et al. (2009) found that children with ASD show a lack of amygdala and FG engagement during emotional face matching, unlike TD children. Similarly, using emotion matching and emotion labelling tasks, children with ASD differentially engaged neural networks, such that they demonstrated attenuated FG and increased precuneus activation during emotion matching, as well as an absence of task-moderated amygdala activity (Wang et al., 2004). Electrophysiological studies of emotion processing have reported attenuated or delayed early (e.g., P100, N170 and N300) and late neural responses in children with ASD (Dawson et al., 2004; Batty et al., 2011; Luckhardt et al., 2017). Using magnetoencephalography (MEG), Leung et al. (2019) demonstrated that children (7–10 years) with ASD show late under-activation in the thalamus and posterior cingulate cortex to happy and angry faces compared to TD peers.

In the last decade, neuroimaging studies in ASD have shifted from examining activation of ‘social brain’ areas independently to examining the function of interconnected networks that support socio-emotional processes. These studies have established atypical patterns of functional connectivity in childhood through to adulthood (Kleinhans et al., 2008; Sato et al., 2012; Khan et al., 2013; Leung et al., 2014; Kana et al., 2016; Mennella et al., 2017; Safar et al., 2018, 2020, 2021). Hypoconnectivity during emotional face processing task-based studies has frequently been reported in various modalities in adults and adolescents with ASD compared to TD (Khan et al., 2013; Leung et al., 2014; Mennella et al., 2017; Safar et al., 2020), while hyperconnectivity has mostly been seen in children (Safar et al., 2018). Using MEG, reduced whole-brain functional connectivity to implicitly presented angry faces was observed in the beta frequency band in adolescents (Leung et al., 2014), and in beta and gamma frequency ranges in adults with ASD (Mennella et al., 2017; Safar et al., 2020), while increased connectivity to happy faces was found in children with ASD in the alpha frequency range (Safar et al., 2018).

Safar et al. (2021) recently established, in a large-sampled study with a broad age range (*n* = 190; 6–39 years), an altered neurodevelopmental trajectory of connectivity in ASD, demonstrating age-related changes in gamma functional connectivity to implicit emotional faces, with connectivity decreasing in ASD, but increasing in TD from childhood to mid-adulthood. Emotion-specific between-group differences with age were also found in the beta band, revealing opposite trajectories of connectivity for happy and angry faces with age in ASD. Specifically, those with ASD showed an age-related decrease in functional connectivity to angry faces, while connectivity to happy faces increased with age. In contrast, the TD group showed an age-related decrease in connectivity to happy faces, while no association with age was found for angry faces. The authors suggested an atypical age-related trajectory of functional connectivity to angry faces in ASD, consistent with impaired age-related proficiency in angry face recognition in this group (Rump et al., 2009), as well as several reports of angry-specific reduced connectivity in adults with ASD (Leung et al., 2014; Mennella et al., 2017; Safar et al., 2020). In addition, age-related increased functional connectivity to happy faces in ASD was thought to reflect difficulty processing happy faces with age, requiring increased compensatory network engagement (Safar et al., 2021).

In addition to classical ADHD symptoms of inattention and hyperactivity/impulsivity (American Psychiatric Association, 2013; Faraone et al., 2021), individuals with ADHD often show social impairments thought to be related to difficulties with emotion recognition and regulation abilities (Shaw et al., 2014). Although less studied than in ASD, converging evidence suggests that emotional face processing atypicalities are common in children, adolescents and adults with ADHD (Brotman et al., 2010; Ibáñez et al., 2011; Ichikawa et al., 2014; Raz and Dan, 2015; Razavi et al., 2017; Flegenheimer et al., 2018; Vandewouw et al., 2020; Ansari Nasab et al., 2021; Viering et al., 2021; Zuberer et al., 2021). Haemodynamic-based approaches have reported attenuated neural activation in emotional face processing tasks in adolescents and adults with ADHD in key brain areas, including the right superior temporal gyrus (STG)/middle temporal gyrus, left amygdala and FG (Viering et al., 2021; Zuberer et al., 2021), while in contrast, children demonstrated increased activation (Brotman et al., 2010; Ichikawa et al., 2014). Similarly, studies using event-related potentials (ERPs) have shown altered neural responses of emotional face processing components, including the P1, N170 and P3 in adults with ADHD, suggesting atypicalities in early and late stages of visual and attentional processing (Ibáñez et al., 2011; Raz and Dan, 2015; Flegenheimer et al., 2018).

Evidence for dysfunctional connectivity to emotional faces has also been recently shown in ADHD (Ansari Nasab et al., 2021; Viering et al., 2021). Using fMRI, Viering et al. (2021) demonstrated that coupling between the right amygdala and ventromedial prefrontal cortex was not modulated by the emotional content of faces in young adults with ADHD, as it was in controls. Furthermore, a sensor-level EEG study in 7-to-11-year-old boys observed increased phase synchronisation in the gamma frequency band among frontal and occipital sites, as well as increased shortest path lengths in occipital-frontal electrode pairs to facial expressions in ADHD compared to TD children (Ansari Nasab et al., 2021). The authors suggested deficits in the efficiency of information transfer in the frontal and occipital lobes in children with the disorder (Ansari Nasab et al., 2021).

Taken together, the above research highlights fundamental differences in neural activation and functional connectivity underpinning emotional face processing in those with ASD and ADHD compared to their TD counterparts. Despite the high degree of symptom overlap among NDD groups, the shared and/or distinct features of functional connectivity underpinning emotional face processing across these two NDDs compared to TD are not well understood. Very few studies have examined the neural mechanisms underlying emotional face processing across these two clinical groups compared to a TD group (Tye et al., 2014; Vandewouw et al., 2020). Vandewouw and colleagues (Vandewouw et al., 2020) used fMRI to investigate differences across youth (5–19 years) with NDDs (ASD, ADHD and obsessive–compulsive disorder; OCD) and TD peers during dynamic emotional face processing and changes in the development of neural responses to faces across the groups. When comparing angry and happy faces (vs. flowers), between-group differences were found in occipital and temporal brain areas. Contrasts revealed that the ASD children showed increased activation to angry vs. happy faces compared to the other NDDs, while no differences were found between TD and ADHD children. When contrasting happy vs. angry faces, age-related differences between NDDs and TD groups were found in the left superior/medial frontal gyri. It was suggested that children with NDDs share neural mechanisms for dynamic face processing that may contribute to difficulties in emotion recognition, in relation to their TD counterparts. It is unknown whether children with NDDs also demonstrate similar functional networks underpinning emotional face processing compared to TD children.

Magnetoencephalography is superbly suited to map the spatial and temporal dynamics of brain-wide networks, as it is a direct measure of neural activity and affords excellent temporal and good spatial resolution (Hari and Salmelin, 2012). MEG has not been previously leveraged to investigate functional connectivity in NDDs and TD individuals; thus, the current MEG study is the first to investigate functional connectivity during the implicit presentation of happy and angry faces in ASD, ADHD and TD groups. Greater difficulties in emotion processing may be seen when tasks are implicit vs. explicit (Wong et al., 2008; Kana et al., 2016; Luckhardt et al., 2017); alterations in neural activation in ASD using implicit emotion processing tasks are widely reported (Critchley et al., 2000b; Batty et al., 2011; Leung et al., 2015, 2018, 2019; Kana et al., 2016; Mennella et al., 2017; Kovarski et al., 2019). Difficulties in implicit emotion processing are thought to be due to the subconscious, rapid and automatic demands, while for explicit processing difficulties may be compensated for by learning strategies, experience and enhanced directed attention to faces (Frith, 2004; Begeer et al., 2006). Thus, we chose to investigate implicit emotion processing, as it taps real-life demands and may be particularly disrupted in NDDs. Additionally, we performed data-driven clustering to determine whether differing patterns of connectivity were discernable among the diagnostic groups. Based on clustering results, measures of functional connectivity strength for significant networks were correlated with behavioural measures. We hypothesised that functional connectivity in children with NDDs would be reduced compared to TD children to happy and particularly angry faces and that different patterns of connectivity would be emotion-dependent. Between-group differences in functional connectivity were expected to be correlated with behavioural measures characteristic of ADHD or ASD, identified by clustering. Due to the overlap in symptoms in ASD and ADHD groups, we further expected that the patterns of connectivity to emotional faces would be more similar in the ASD and ADHD groups compared to TD controls.

## Materials and Methods

### Participants

The original cohort of participants included 395 children 5–19 years of age with ADHD, ASD and age- and sex-matched controls: 111 with ADHD, 176 with ASD and 108 TD controls. Participants were recruited through the Province of Ontario Neurodevelopmental Disorders (POND) network. All children underwent MEG scanning between 2012 and 2020. Primary diagnoses of ADHD and ASD were assigned by expert clinicians and confirmed with the Parent Interview for Child Symptoms (PICS; Ickowicz et al., 2006) for ADHD, and the Autism diagnostic Observation Schedule-2 (Lord et al., 2012) and Autism Diagnostic Interview-Revised (Lord et al., 1994) for ASD. Potential TD participants were not included if they were born premature or were diagnosed with learning, language, neurological or developmental disabilities. The study protocol was approved by the Hospital for Sick Children and Holland Bloorview Research Ethics Boards. A parent or legal guardian of all child participants granted written informed consent; all child participants gave informed verbal assent.

Full-scale IQ (FSIQ) was measured using Wechsler scales of intelligence (Wechsler, 1999, 2003, 2012, 2014). To capture behaviours characteristic of ADHD or ASD yet expressed in the other diagnosis, the Child Behaviour Checklist attention problems subscale (CBCL-AP; Achenbach and Rescorla, 2001) was used to measure inattention, and the Social Communication Questionnaire total score (SCQ-TOT; Berument et al., 1999) was used to measure social communication problems. To measure difficulties in adaptive functioning observed in both disorders, the Adaptive Behaviour Assessment System’s (Harrison and Oakland, 2003) General Adaptive Composite score (ABAS-GAC) was used.

### Implicit Emotional Face Processing Task

**Figure 1:**
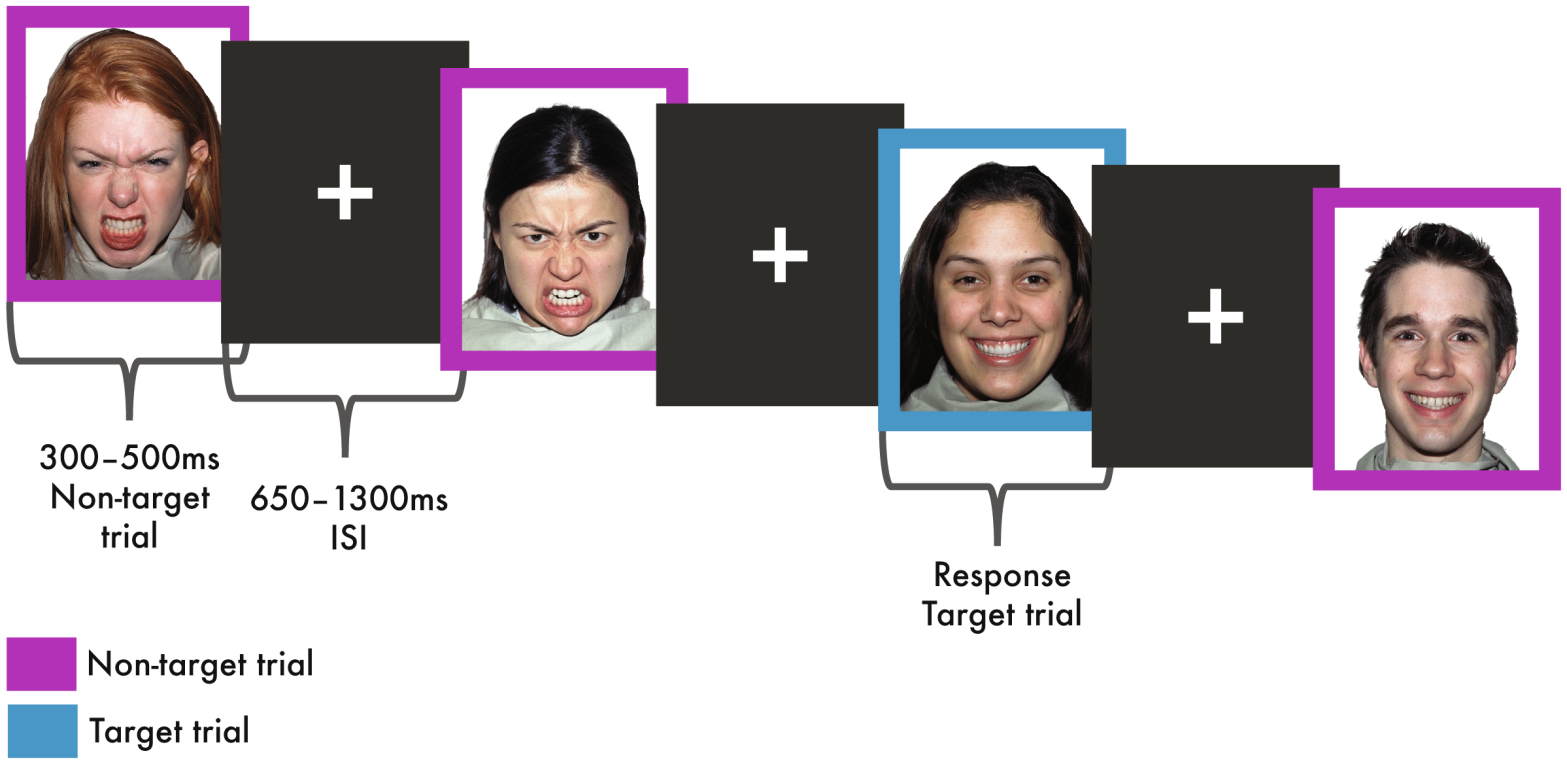
Implicit emotional faces task. During all the trials, participants attended to the colour of the border and ignored the emotional content of the faces. The non-target trials comprised 75% of the trials (shown here in purple). Target or ‘catch’ trials were presented randomly, comprising 25% of trials (shown here in blue). During target trials, participants rapidly responded to their assigned border colour, in this example blue, using a button-press. Faces were extracted from the MacBrain Face Stimulus Set (http://www.macbrain.org/resources.htm), the actors presented here have been previously published (Tottenham et al., 2009).

The implicit emotional face processing task consisted of randomly presented happy and angry faces (52 faces and 12 female) extracted from the MacBrain Face Stimulus Set^1^ (Tottenham et al., 2009). The emotional faces were each formatted to a size of 7.4w × 9 h cm with a 2 cm blue or purple border surrounding the image. Presentation^®^ software^2^ was used to present the task, with the emotional face stimuli subtended ~14 × 16 degrees of visual angle, from a viewing distance of 78 cm. The faces were presented rapidly, while participants concentrated on the colour of the border and ignored the emotional content of the faces; these non-target trials comprised 75% of total trial counts. Randomly presented target or ‘catch’ trials were included to ensure that participants were attending to the task; these trials comprised the other 25% of trials and were identified by the colour of the border. For the target trials, participants responded to their assigned border colour (either blue or purple) as quickly as possible using a MEG-compatible button-press, also still ignoring the emotional content of the faces. The assigned border colour was counter-balanced across participants. Each trial consisted of a happy or angry face, with the duration adjusted between 300 and 500 ms to maintain consistent error rates (≥95% for target trials, ≥80% for non-target trials) and followed by a 650–1,300 ms inter-stimulus interval (ISI; fixation cross) also adjusted according to error rates (Figure 1). Reaction times to the emotional faces for target trials were recorded for behavioural analysis; however, only correct non-target trials were used for MEG analyses to avoid motor activity associated with the button-press for the target trials. Participants’ data were excluded from analyses if accuracy on the target trials or the non-target trials was less than chance (<55%).

### MRI and MEG Data Acquisition

Participants completed the task in the supine position in a magnetically shielded room while MEG data were recorded using a 151-channel CTF system (CTF MEG International Services LP, Coquitlam, BC, Canada). Data were sampled at 600 Hz with an online 0–150 Hz antialiasing filter, and a third-order spatial gradient applied to reduce environmental noise. Head location was continuously monitored using fiducial coils that were fitted at the left and right pre-auricular and nasion. Following scanning, the fiducial coils were replaced with radio-opaque markers for MRI co-registration. For co-registration, individual structural T1-weighted images were acquired on a Siemens 3.0 T MAGNETOM Trio with a 12 channel head coil (TR/TE = 2300/2.96 ms, FA = 9°, FOV = 240×256 mm, # slices = 192, resolution = 1.0 mm isotropic) scanner or, due to a scanner upgrade during the study, on a PrismaFIT with a 20 channel head and neck coil (TR/TE = 1870/3.14 ms, FA = 9°, FOV = 240×256 mm, # slices = 192, resolution = 0.8 mm isotropic) scanner.

### MEG Preprocessing and Source Reconstruction

The FieldTrip toolbox (Oostenveld et al., 2011) in MATLAB (The Mathworks Inc., 2018) was used for MEG data preprocessing and source reconstruction. The MEG data were band-pass filtered between 1 and 150 Hz using a 4th order two-pass Butterworth filter, and line noise was removed from the signal at 60 and 120 Hz using a discrete Fourier transform notch filter. The data were epoched into −1,000–1,250 ms happy or angry trials, relative to the onset of the emotional face stimuli. We applied independent component analysis (ICA) to attenuate heartbeat or ocular (i.e., saccades and blinks) artefacts contaminating the MEG signal; these components were manually removed. After ICA, trials were excluded from analyses if the sensor signals exceeded 2000 ft., or if the initial median head location was shifted greater than 10 mm [a threshold recommended for developmental populations (Pang, 2011; Safar et al., 2018)]. Data from participants with greater than 20 trials remaining after artefact rejection, in each emotion category, were retained for statistical analyses.

To co-register the MEG data, each participant’s anatomical T1-weighted MRI was used to generate an individual single-shell head model (Nolte, 2003). The centroids of the first 90 parcels of the Automated Anatomical Labelling (AAL) atlas (Tzourio-Mazoyer et al., 2002) including subcortical and cortical source locations were non-linearly warped into analogous subject-specific head locations from standard template space (ICBM 152; Fonov et al., 2011). To estimate the broadband time series of source activity for each of the 90 AAL parcels, a vector (LCMV) beamformer (van Veen et al., 1997), with 5% Tikonov regularisation, was applied. The neural activity index (NAI) was then computed by normalising the amplitudes of the source reconstructed time series data by the estimated amplitude of projected noise to eliminate the centre-of-head bias (van Veen et al., 1997).

### Functional Connectivity

The broadband time series data at each of the 90 AAL sources were filtered into theta (4–7 Hz), alpha (8–14 Hz), beta (15–29 Hz) and gamma (30–55 Hz) canonical frequency bands. The time series of instantaneous phase values at each source and frequency were then obtained by computing the Hilbert Transform, and the phase data were epoched from −400 to 600 ms, relative to stimulus onset. Instantaneous phase synchrony at each sample across the time series between pairwise sources was estimated using the cross-trial phase lag index (PLI) based on Stam et al. (2007). The PLI measures the asymmetry in the distribution of phase differences between two source signals (i.e., lags and leads), while correlated sources with zero or near zero phase lags are accounted for, and thus, the possibility of spurious pairwise interactions are attenuated (i.e., volume conduction from one strong source). Whole-brain PLI was computed pairwise between each of the 90 AAL sources; this yielded a 90-by-90 adjacency matrix for each sample across the timeseries, within each emotion category and frequency range, for each participant. The time window selected for statistical analyses was 200–400 ms after stimulus onset and the relative change from the baseline window (−200–0 ms) was calculated for the PLI values and averaged across this time window. The time window of interest was selected based on previous research from our group (Mennella et al., 2017; Safar et al., 2021) demonstrating sensitivity to differences in implicit emotional face processing in ASD compared to controls at this latency window.

### Statistical Analyses

Differences among the diagnostic groups in demographics were assessed with one-way ANOVAs for the continuous variables (age, head motion, number of trials and the behavioural measures), with significance held at *p* < 0.05; upon significance, Bonferroni-adjusted significance tests for pairwise comparisons were performed, holding significance at *p*_corr_ < 0.05. A chi-squared test was used to investigate differences in the proportion of males and females (*p* < 0.05). A repeated-measures ANOVA with emotion (happy and angry) as the within-group factor and diagnosis (ADHD, ASD and TD) as the between-group factor was used to ensure no significant effects of emotion, group, nor their interaction on the number of trials used in the analyses, accuracy and reaction time to the target and accuracy to the non-target trials; significance was held at *p* < 0.05.

For functional connectivity analyses, an ANCOVA within Network-Based Statistics (NBS; Zalesky et al., 2010, 2012) was used. First, for each frequency band, the main effect of each emotion (happy and angry) compared to the baseline fixation cross was extracted for the data-driven subgrouping (see next section). Next, we tested the main effect of group on functional connectivity, with age and sex as covariates, the main effect of emotion and the group-by-emotion interaction, for each frequency band. Additionally, we tested for group-by-age and group-by-sex interactions, as well as emotion-by-age and emotion-by-sex interactions. The NBS is a well-established non-parametric statistical method optimal for the analysis of large networks, which accounts for the family-wise error rate (FWER; Zalesky et al., 2010, 2012).^3^ The primary component-forming thresholds were chosen based on the sparsity of the networks, such that the networks comprised 1% of total possible network connections. In all analyses, 5,000 permutations were conducted.

### Data-Driven Subgrouping

From the main effects of emotion compared to the baseline (across diagnostic groups) for each frequency band, the mean network strength was extracted (eight measures of network strength from 2 emotions × 4 frequency bands) and used in a data-driven subgrouping procedure implemented in MATLAB (The Mathworks Inc., 2018). For a pre-specified number of subgroups, *k*, 5,000 bootstrap iterations were performed. For each iteration, 63.2% (Strobl et al., 2007) of the participant sample was selected, and *k*-means clustering (Lloyd, 1982) with the Euclidean distance metric was used to identify *k* subgroups of participants from the eight measures of mean network strength. For each *k*, the bootstrap iterations were amalgamated by computing a participant consensus matrix whose entries contain the percentage of times pairs of participants were placed in the same subgroup; the participant consensus matrix was then partitioned using spectral clustering. The number of subgroups, *k*, was varied between 2 and 10, and the optimal number of subgroups was evaluated using the Calinski-Harabasz index (Caliñski and Harabasz, 1974). Differences among the identified subgroups in age, behavioural measures (FSIQ, CBCL-AP, SCQ-TOT and ABAS-GAC) and eight mean network strengths were examined using one-way ANOVAs, holding significance at *p* < 0.05, with Bonferroni-adjusted significance tests for pairwise comparisons upon significance, with *p*_corr_ < 0.05. Differences among the identified subgroups in sex and diagnosis were examined using chi-squared tests (*p* < 0.05).

### Brain-Behaviour Correlations

Based on clustering results, measures of network strength for significant networks were correlated with behavioural measures identified by the clustering, in this study, CBCL-AP and ABAS-GAC.

## Results

### Participant Demographics

The analysis included 258 children 5–19 years of age with ADHD, ASD and age- and sex-matched controls: 71 with ADHD, 100 with ASD and 87 TD controls. Of the initial 395 participants, 137 were excluded due to (a) < 20 clean MEG trials in each emotion condition (*n* = 69); (b) < 55% task accuracy (*n* = 6); (c) poor head localization (*n* = 16); and a further *n* = 46 were excluded due to participant matching on age, sex and head motion. FSIQ, CBCL-AP, SCQ-TOT and ABAS-GAC were collected on 226, 180, 180 and 171 of the participants, respectively. Participant demographics are summarised in Table 1.

**Table 1:** Participant demographics.

		ADHD	ASD	TD
*N*		71	100	87
Age (years; mean ± std.)		11.55 ± 2.57	12.22 ± 3.20	11.28 ± 3.50
Age range		6.51–18.18	6.78–19.00	5.90–19.91
Sex (M:F)		54:17	77:23	55:32
Mean head motion (mm; mean ± std.)		0.84 ± 0.45	0.81 ± 0.41	0.78 ± 0.52
Number of trials (mean ± std.)	Happy	35.97 ± 5.95	36.67 ± 4.54	37.51 ± 2.99
	Angry	35.41 ± 6.12	37.22 ± 4.04	36.90 ± 3.77
FSIQ (mean ± std.)		102.92 ± 13.21	100.12 ± 16.55	112.10 ± 12.72
CBCL-AP (mean ± std.)		93.51 ± 6.84	89.88 ± 11.55	56.32 ± 9.57
SCQ-TOT (mean ± std.)		7.19 ± 5.25	19.31 ± 7.03	2.45 ± 2.74
ABAS-GAC (mean ± std.)		82.64 ± 14.10	70.01 ± 14.04	102.16 ± 14.18

*FSIQ, Full-scale IQ; CBCL-AP, Child Behavioural Checklist attentional problems; SCQ-TOT, Social Communication Questionnaire total score; and ABAS-GAC, Adaptive Behaviour Assessment System General Adaptive Composite score*.

There were no significant differences in age [*F*(2, 255) = 2.216, *p* = 0.11] or in the proportion of boys and girls (*χ*^2^ = 5.16, *p* = 0.08) between diagnostic groups. No significant between-group differences were observed in head movement [*F*(2, 255) = 0.28, *p* = 0.76].

Furthermore, there was no difference in the number of trials included in the analysis between diagnostic groups [*F*(2, 255) = 2.89, *p* = 0.057] or in the number of happy compared to angry trials included [*F*(1, 255) = 0.73, *p* = 0.40], nor was there a group-by-emotion interaction [*F*(2, 255) = 2.65, *p* = 0.073].

The diagnostic groups significantly differed in FSIQ [*F*(2, 223) = 15.15, *p* = 6.80 × 10^−7^], CBCL-AP [*F*(2, 177) = 204.45, *p* = 9.83 × 10^−47^], SCQ-TOT [*F*(2, 177) = 139.01, *p* = 5.13 × 10^−37^] and ABAS-GAC [*F*(2, 168) = 64.24, *p* = 1.90 × 10^−21^]. Pairwise Bonferroni-adjusted post-hoc tests revealed that the TD participants had significantly higher FSIQ compared to both diagnostic groups (ADHD: *p*_corr_ = 7.12 × 10^−4^, ASD: *p*_corr_ = 7.09 × 10^−7^), while the ADHD and ASD participants showed no difference in FSIQ (*p* = 0.74). The TD participants also showed significantly fewer attention problems (CBCL-AP) compared to both diagnostic groups (ADHD: *p*_corr_ = 2.88 × 10^−44^, ASD: *p*_corr_ = 4.36 × 10^−40^), who did not differ from one another (*p*_corr_ = 0.08). For SCQ-TOT and ABAS-GAC, compared to the TD participants, both the ADHD (SCQ-TOT: *p*_corr_ = 1.21 × 10^−4^; ABAS-GAC: *p*_corr_ = 1.19 × 10^−9^) and ASD (SCQ-TOT: *p*_corr_ = 3.07 × 10^−33^; ABAS-GAC: *p*_corr_ = 6.35 × 10^−22^) participants showed greater difficulties in social communication and adaptive functioning, with the ASDs also showing greater difficulties compared to the ADHDs (SCQ-TOT: *p*_corr_ = 3.80 × 10^−26^; ABAS-GAC: *p*_corr_ = 2.02 × 10^−6^).

### Task Accuracy and Reaction Time

For the target trials, we found no significant main effects of group, [*F*(2,255) = 1.83, *p* = 0.162], or emotion [*F*(1,255) = 2.24, *p* = 0.136], and no emotion-by-group [*F*(2,255) = 2.25, *p* = 0.108] interaction for accuracy. Similarly, for reaction time, there were no significant main effects of group [*F*(2,255) = 2.26, *p* = 0.106], or emotion [*F*(1,255) = 1.315, *p* = 0.253], and no emotion-by-group [*F*(2,255) = 1.869, *p* = 0.156] interaction found.

For the non-target trials, no significant main effect of group [*F*(2,255) = 1.224, *p* = 0.296], was observed for accuracy (i.e., not responding); however, a main effect of emotion was found [*F*(1,255) = 5.737, *p* = 0.017], such that accuracy was greater for happy compared to angry trials across groups. No emotion-by-group [*F*(2,255) = 0.393, *p* = 0.675] interaction was found for accuracy. Task accuracy and reaction time are summarised in Table 2.

**Table 2:** Task accuracy and reaction time.

			ADHD	ASD	TD
*N*			71	100	87
Accuracy (%)	Target trials (mean ± std.) Non-target trials (mean ± std.)	Happy Angry Happy Angry	93.25 ± 8.25 91.25 ± 11.41 95.64 ± 5.95 94.85 ± 7.06	93.96 ± 8.01 94.78 ± 6.92 96.73 ± 5.40 96.43 ± 6.19	93.30 ± 9.64 91.81 ± 11.58 96.10 ± 5.51 95.61 ± 5.05
Reaction time (ms)	Target trials (mean ± std.)	Happy Angry	273.17 ± 56.67 274.05 ± 65.67	260.85 ± 65.29 260.77 ± 61.33	285.10 ± 80.32 277.64 ± 78.03

### Functional Connectivity

**Figure 2:**
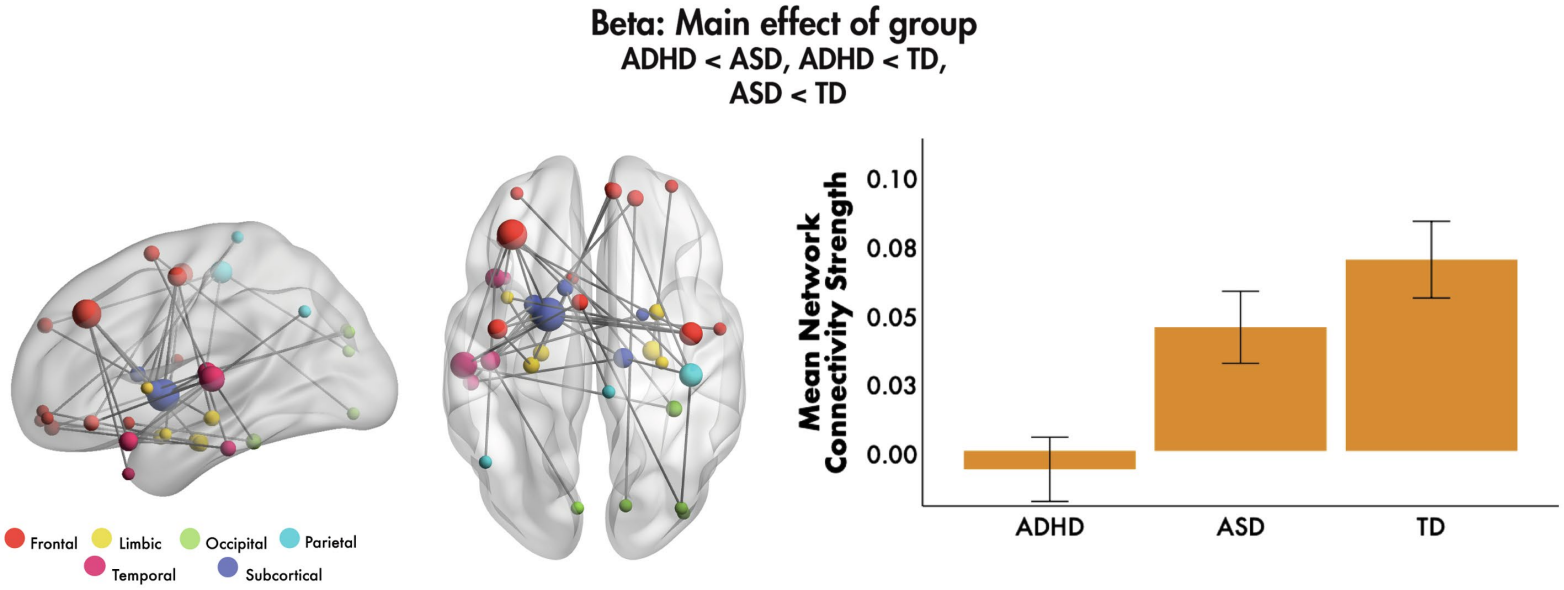
Main effect of group, 200–400 ms to emotional faces, following the onset of non-target trials. A significant main effect of group was observed in the beta frequency range to emotional faces. The glass brains represent the network, where node size is scaled by degree. The mean network connectivity strength is plotted for each group in the bar graph (error bars represent 95% confidence intervals).

No significant main effects of emotion were found, nor emotion-by-age or emotion-by-sex interactions (all *p_corr_* > 0.05, FWER-corrected). We found a main effect of group in the beta frequency band, with covariates age and sex (*F* = 4.85, 40 edges, 37 nodes, *p_corr_* = 0.007, Figure 2). *Post-hoc* tests showed that mean network connectivity strength was reduced in ADHD compared to ASD (*p_corr_* < 0.001) and TD (*p_corr_* < 0.001) groups, and in ASD compared to the TD group (*p_corr_* = 0.021; all Bonferroni-corrected for multiple comparisons). The network primarily involved frontal, subcortical and temporal connections, mostly anchored in the left hemisphere, with the most highly connected being the left middle frontal gyrus, pallidum and STG. The network also involved key face processing areas including the bilateral amygdalae, right FG and left insula, as well as orbital frontal areas with connections to limbic, occipital, parietal and temporal brain areas. No significant main effects of group were found in the theta, alpha or gamma bands (all *p_corr_* > 0.05).

**Figure 3:**
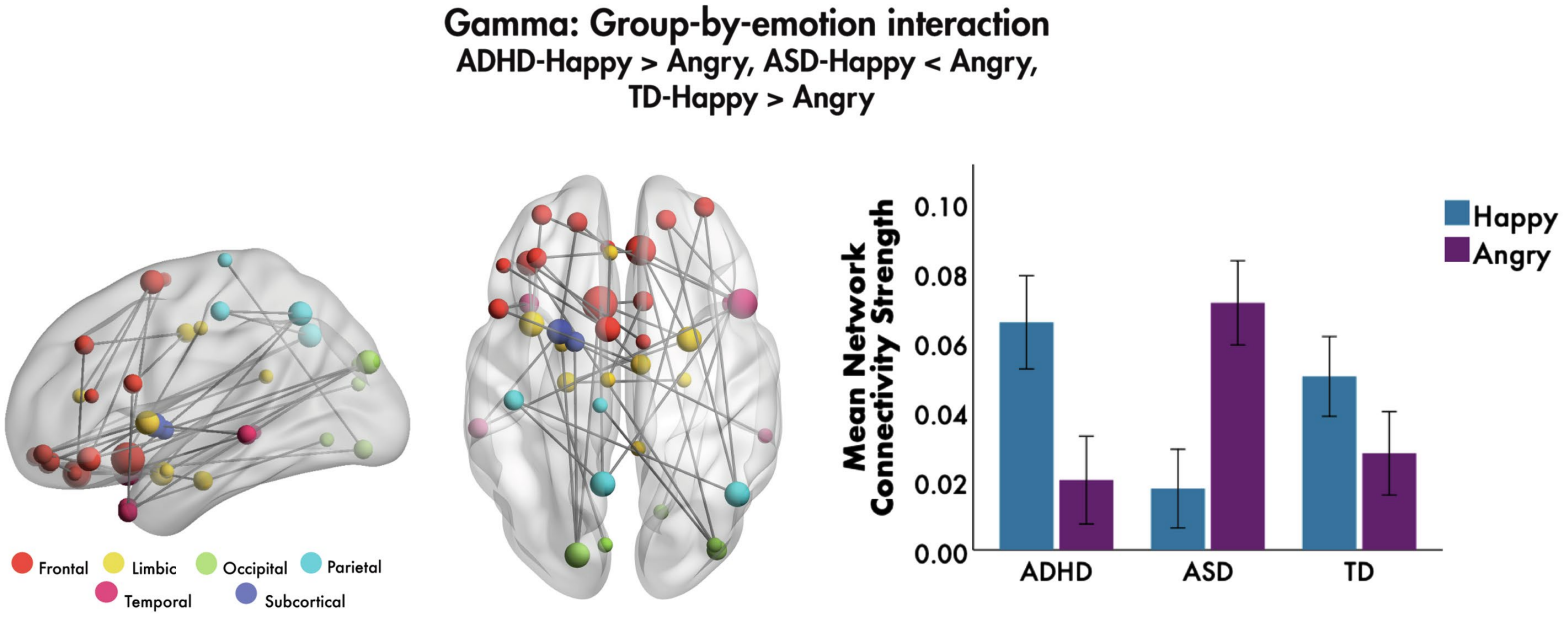
Group-by-emotion interaction, 200–400 ms to emotional faces, following the onset of non-target trials. A significant group-by-emotion interaction was seen in the gamma band to happy and angry faces, indicating that functional connectivity network strength in each group is modulated by the valence of the emotional faces. The glass brains represent the network, where node size is scaled by degree. The mean network connectivity strength for this network by emotion is plotted for each group in the bar graph (error bars represent 95% confidence intervals).

The only group-by-emotion interaction was found in the gamma frequency band (*F* = 4.7, 40 edges, 39 nodes, *p_corr_* = 0.026, Figure 3). A follow-up simple effects analysis on the mean network connectivity strength was performed to compare functional connectivity to happy and angry faces in each of the groups. Those with ADHD showed significantly greater connectivity to happy compared to angry faces, *F*(1, 255) = 22.59, *p_corr_* < 0.001, as did those in the TD group, *F*(1, 255) = 6.58, *p_corr_* = 0.033; while those with ASD demonstrated the opposite pattern, such that connectivity was significantly greater to angry relative to happy faces, *F*(1, 255) = 44.08, *p_corr_* < 0.001. The network involved connections among bilateral frontal, largely inferior and orbital frontal, along with limbic and temporal brain areas. Most connections were among frontal and limbic areas, including the right amygdala, left insula and left anterior cingulate cortex (ACC). Bilateral superior and middle orbital frontal and left inferior frontal regions were connected to the right superior and left middle temporal pole, respectively; connections between right orbital frontal areas and the right angular gyrus were also seen. No group-by-age or group-by-sex interactions were found in the theta, alpha, beta or gamma bands (all *p_corr_* > 0.05).

### Data-Driven Subgrouping

The main effects of each emotion compared to baseline (across diagnostic group) in each frequency band were used in a data-driven subgrouping procedure. The Calinski-Harabasz index showed that the optimal number of subgroups was two. The first subgroup, Subgroup-1, consisted of 28 ADHD, 38 ASD and 22 TD participants, while the second, Subgroup-2, consisted of 43 ADHD, 62 ASD and 65 TD. Subgroup demographics are summarised in Table 3.

**Table 3:** Subgroup demographics.

	Subgroup-1 (NDD-dominant)	Subgroup-2 (TD-dominant)
*N*	88	170
Diagnosis (ADHD:ASD:TD)	28:38:22	43:62:65
Age (years; mean ± std.)	11.64 ± 3.17	11.76 ± 3.17
Sex (M:F)	66:22	120:50
FSIQ (mean ± std.)	102.25 ± 14.91	106.46 ± 15.34
CBCL-AP (mean ± std.)	89.31 ± 13.23	81.29 ± 18.75
SCQ-TOT (mean ± std.)	12.25 ± 9.68	10.27 ± 8.57
ABAS-GAC (mean ± std.)	77.40 ± 16.39	83.68 ± 19.41

*FSIQ, Full-scale IQ; CBCL-AP, Child Behavioural Checklist attentional problems; SCQ-TOT, Social Communication Questionnaire total score; and ABAS-GAC, Adaptive Behaviour Assessment System General Adaptive Composite score*.

There were no significant differences between the subgroups in age [*F*(1, 256) = 0.09, *p* = 0.77] nor sex (*χ*^2^ = 0.56, *p* = 0.45). While the proportion of the diagnoses did not differ between the subgroups (*χ*^2^ = 4.58, *p* = 0.10), there was a significantly different proportion of TD and NDD participants between the subgroups (*χ*^2^ = 4.54, *p* = 0.03), with Subgroup-1 consisting of a higher proportion of NDD and lower proportion of TD participants, and Subgroup-2 consisting of a higher proportion of TD and a lower proportion of NDD participants; thus, Subgroup-1 is termed the NDD-dominant subgroup and Subgroup-2 the TD-dominant subgroup.

There was no significant difference between the subgroups in FSIQ [*F*(1,224) = 3.83, *p* = 0.05] and SCQ-TOT [*F*(1,178) = 2.01, *p* = 0.16], although differences in FSIQ were approaching significance with the TD-dominant subgroup having higher FSIQ than the NDD-dominant subgroup. Significant differences between the subgroups were observed in CBCL-AP [*F*(1,178) = 9.17, *p* = 2.82 × 10^−3^] and ABAS-GAC [*F*(1,169) = 4.52, *p* = 0.03], with the NDD-dominant subgroup having more attention and adaptive functioning problems compared to the TD-dominant subgroup.

**Figure 4:**
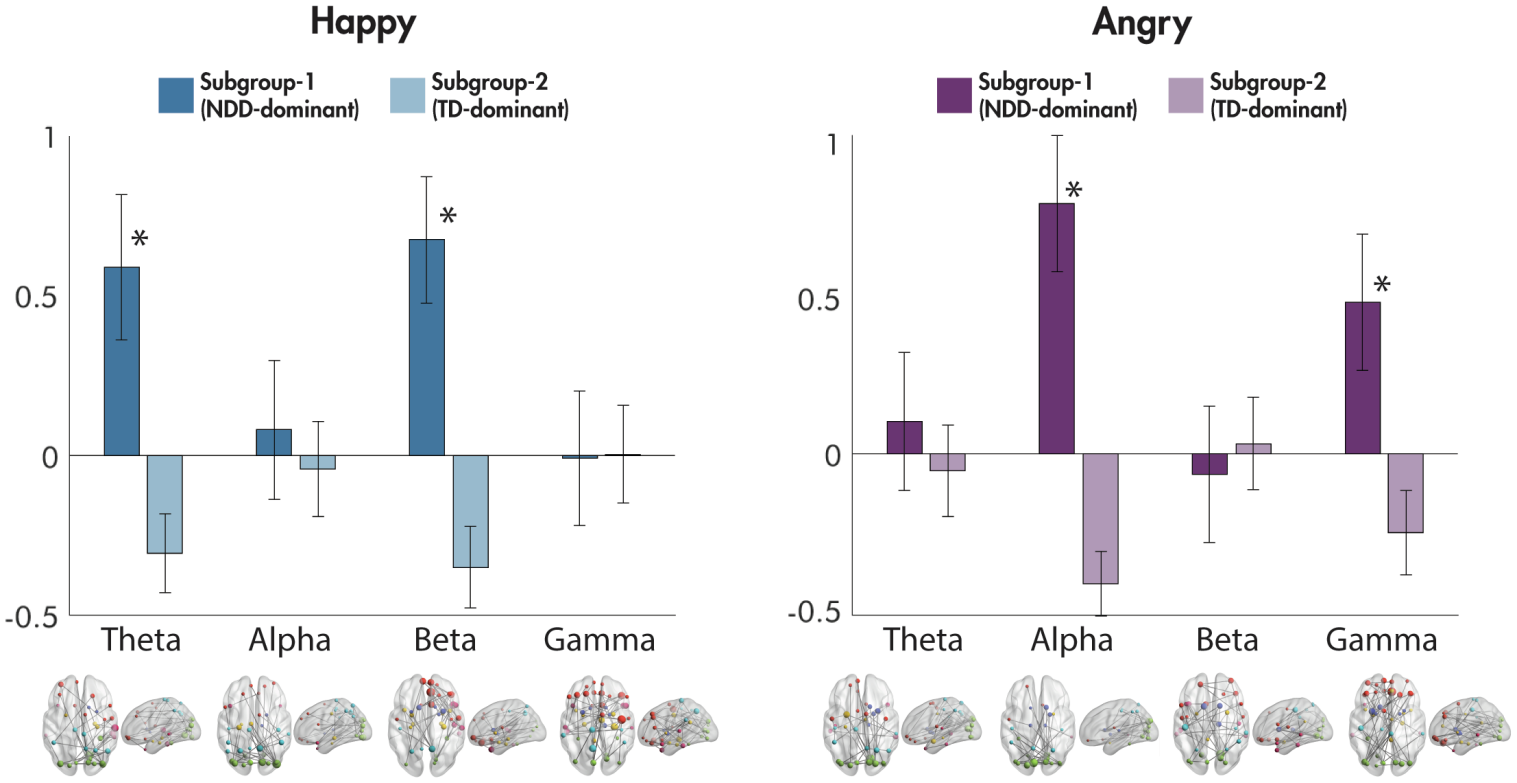
Differences between subgroups in mean network connectivity strength for happy and for angry faces compared to baseline. The mean network connectivity strength for each significant network to happy and angry faces compared to baseline for each frequency band is plotted for each of the subgroups in the bar graphs (error bars represent standard deviation). Significant networks are plotted in the glass brains under each corresponding frequency range. ^*^p < 0.001.

Differences between the subgroups in the eight mean network strengths used as an input to the data-driven subgrouping were also examined (Figure 4). For happy faces, significant differences were observed in the theta [*F*(1,256) = 56.16, *p* = 1.09 × 10^−12^] and beta [*F*(1,256) = 79.37, *p* = 9.84 × 10^−17^] frequency bands, with the NDD-dominant subgroup showing an increase in the mean network strength compared to baseline, and the TD-dominant subgroup showing a decrease; no differences were observed in the alpha [*F*(1,256) = 0.86, *p* = 0.35] nor gamma [*F*(1,256) = 0.01, *p* = 0.92] bands. In contrast, for angry faces, significant differences between the subgroups in mean network strength were found in alpha [*F*(1,256) = 129.05, *p* = 1.74 × 10^−24^] and gamma [*F*(1,256) = 36.06, *p* = 6.52 × 10^−9^], where, again, the NDD-dominant subgroup showing an increase in the mean network strength compared to baseline, and the TD-dominant subgroup showing a decrease; no significant differences existed in the theta [*F*(1,256) = 1.44, *p* = 0.23] nor beta [*F*(1,256) = 0.56, *p* = 0.45] frequency bands.

### Brain-Behaviour Correlations

Brain-behaviour correlations were performed between measures of network strength for the main effect of group in the beta band and the group-by-emotion interaction in the gamma band with the CBCL-AP and ABAS-GAC, respectively. We correlated network strength in the beta range with the CBCL-AP to determine whether difficulties in attention were associated with between-group differences in connectivity and subgroups. The CBCL-AP was chosen as the ADHD group was noticeably different from the other groups on the connectivity metric, and it is the CBCL that is diagnostic of the behavioural differences. Additionally, we correlated network strength in the gamma range to angry and happy faces with the ABAS-GAC to establish whether adaptive behavioural functioning was related to connectivity. Again, the ABAS was selected as the ASD group differed considerably from other groups, and the ABAS reflects adaptive behavioural symptoms.

**Figure 5:**
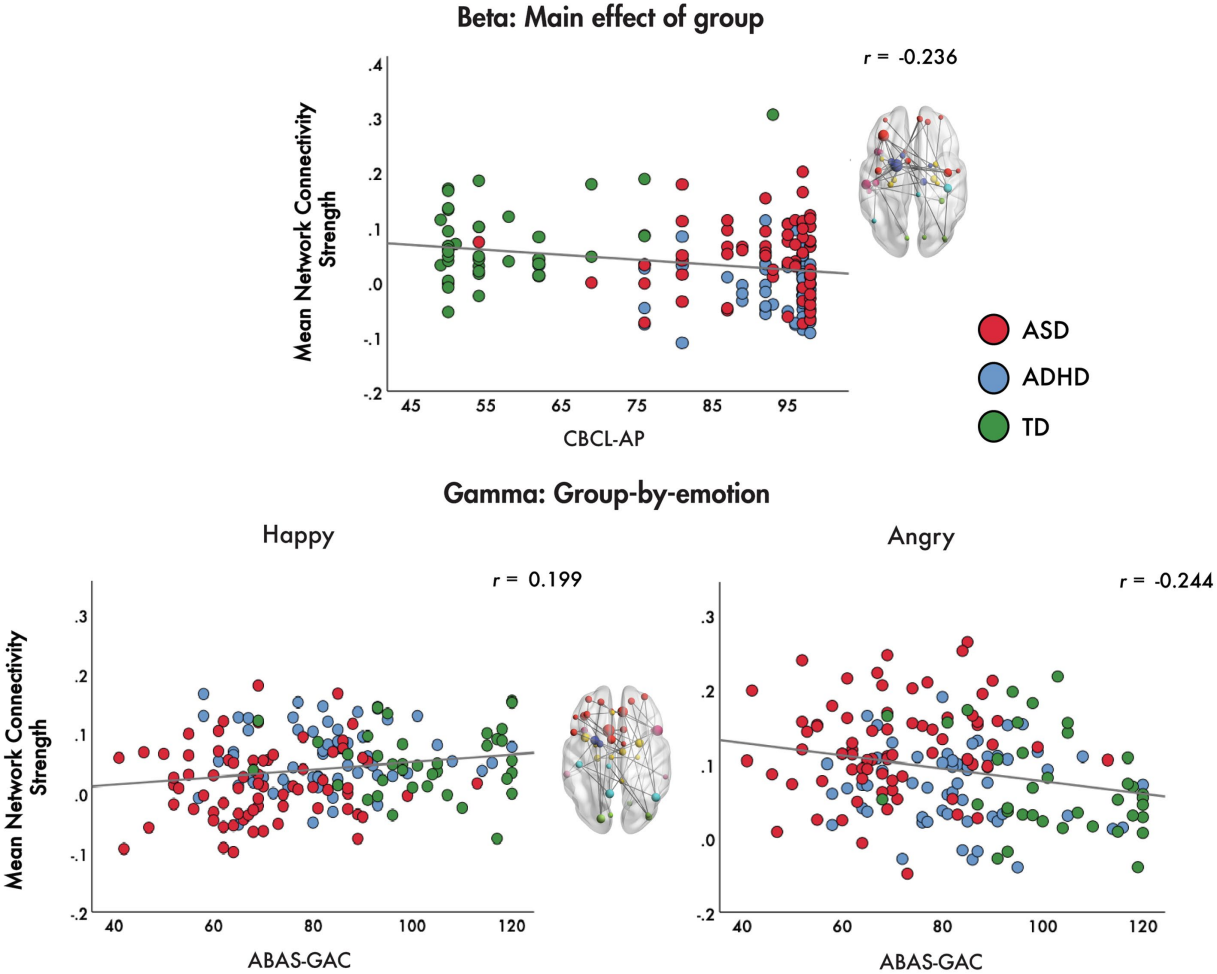
Brain-behaviour correlations. Brain-behaviour correlations are plotted for the mean network connectivity strength for the main effect of group in the beta band and the group-by-emotion interaction in the gamma band with the CBCL-AP and ABAS-GAC, respectively.

For the main effect of group, we found that mean network connectivity strength was negatively correlated with CBCL-AP (*r* = −0.236, *p* = 0.001) across the ADHD, ASD and TD groups. In the gamma band, for the group-by-emotion interaction, the mean network connectivity strength to angry faces was negatively correlated with ABAS-GAC scores (*r* = −0.244, *p* = 0.001), while connectivity strength to happy faces was positively correlated with ABAS-GAC scores (*r* = 0.199, *p* = 0.009) across the groups (Figure 5).

## Discussion

In this study, we capitalised on the superb resolution of MEG in the temporal, spatial and oscillatory domains to identify distinct patterns of functional connectivity between ASD, ADHD and controls on an emotional face processing task. With the large sample size in this study, we were also able to apply data-driven clustering methods to determine that ASD and ADHD children as NDDs share similarities in underpinning neural mechanisms. Further, brain-behaviour correlations demonstrated that atypicalities in specific brain networks were significantly correlated with the degree of severity of diagnosis-specific symptom characteristics lending strong support to the idea that specific brain networks may underlie symptom presentation and severity.

In the beta band, a main effect of group was observed where ASD showed significantly reduced functional connectivity compared to controls. Surprisingly, the ADHD group showed a further significant reduction in connectivity in a left hemisphere predominant network involving frontal, subcortical and temporal connections which included nodes in dorsolateral prefrontal cortex (dlPFC), pallidum and STG. However, a significant negative correlation between decreased strength of this network with poorer scores on the attention subscale of the CBCL may explain this finding, as the dlPFC is known to be involved in attentional control (Rossi et al., 2009) and known to be a deficit in ADHD (Cubillo et al., 2012).

Particularly interesting was the reduced connectivity between prefrontal areas and the pallidum, a region known to play a key role in driving reward and motivation behaviour (Smith et al., 2009; Arsalidou et al., 2013), especially with regards to emotional regulation (Arnsten and Rubia, 2012; Brandl et al., 2019). Our finding of reduced connectivity in the beta band further supports that the ADHD group may not find an emotion processing task rewarding. More recent evidence proposes that increased beta activity is a brain signature of a positive reward (Marco-Pallarés et al., 2015). Our findings suggest that poor attentional control may be related to reduced perception of reward, and therefore, lower motivation, for this emotion processing task in ADHD, consistent with previous literature (Van Hulst et al., 2017; Sali et al., 2018).

In the gamma band, as expected for the TD group, we observed a pattern of connectivity in a network known to be involved in emotion processing, which included orbital frontal and limbic regions (Pessoa, 2017). We also observed greater connectivity strength for happy compared to angry faces in the TD group, also as expected from the extensive literature showing a happy face advantage (e.g., Kirita and Endo, 1995) in that happy faces are preferred, engaging, invitational and approachable (e.g., Becker et al., 2011; Nikitin and Freund, 2019). The ADHD group showed a similar pattern to the TD; however, the ASD showed the opposite effect with angry faces inducing greater connectivity in this network than happy faces. Given the considerable evidence for impairments in the recognition of facial expressions and inappropriate responses to facial affect that are associated with ASD (for a meta-analytic review, see Lozier et al., 2014), this was not surprising. In particular, prior studies examining differences in functional connectivity in ASD report connectivity change trajectories moving in opposite directions for ASD compared to controls for angry and happy faces (e.g., Mamashli et al., 2018), with greater network connectivity strength for angry compared to happy faces and the reverse developmental trajectory compared to TD (Safar et al., 2021). This is further supported by our finding of a significant positive correlation between network connectivity strength for happy faces and ABAS-GAC scores, a measure of general adaptive functioning. Thus, lower adaptive functioning, a domain in which those with ASD are known to have deficits (Lopata et al., 2012), was correlated with lower connectivity strength of this happy network, which we observed in the ASD group.

Our finding of a significant negative correlation between network strength for angry faces and scores on the ABAS-GAC was very interesting. This shows that higher network connectivity to angry faces is related to lower adaptive functioning (often seen in the ASD group) while lower network connectivity to angry is related to higher adaptive functioning, as seen in the TD and ADHD groups. This would seem counter-intuitive except that the gamma band synchronisation is thought to play a fundamental role in cortical computation (Fries, 2009) by regulating and modulating information transfer (Sohal, 2016). Excessive gamma connectivity to angry faces in the ASD group may suggest that they allocate atypical and excessive resources to the regulation and modulation of angry face stimuli, perhaps leaving fewer resources to allocate to other tasks, with the result being insufficient resources for use in the processing of more appropriate and adaptive tasks. This hypothesis remains to be directly tested.

Most striking were the data-driven clustering results from this study. Despite the discussion above describing the differences between ASD and ADHD, the data are clear that both groups share more similarities as NDDs than they differ as distinct diagnoses. Our findings add to an accumulating body of literature corroborating the high degree of comorbidity and symptom overlap seen in the various NDDs (Ronald et al., 2008; Kushki et al., 2019; Lai et al., 2019), pointing towards the existence of shared biological mechanisms. Our study is also in line with fMRI findings of shared neural mechanisms in children with NDDs for a dynamic face processing task (Vandewouw et al., 2020). Finally, our brain-behaviour correlations reinforce this idea as symptom severity on diagnosis-specific measures correlate strongly with metrics of brain network connectivity. Given the growing body of literature highlighting shared underlying neurobiology among NDDs using data-driven methods (Kushki et al., 2019, 2021; Choi et al., 2020; Vandewouw et al., 2021), these approaches may facilitate an understanding of the similarities and individual differences among these disorders, without reliance on diagnostic categories. In terms of intervention, diagnostic status may be an inadequate indicator of the broader needs of children with NDDs and not fully capture behavioural and cognitive sequalae (Kushki et al., 2019; Astle et al., 2021). Thus, taking a transdiagnostic child needs-based approach rather than focus on diagnosis-specific primary deficits may better serve to support children with NDDs (Astle and Fletcher-Watson, 2020; Astle et al., 2021; Kushki et al., 2021).

Given the importance of understanding facial expressions for the appropriate development of social communication skills, the results reported in this study suggest that ADHD differs from ASD in that children with ADHD do not allocate sufficient attention to the task of processing faces, regardless of emotion. On the other hand, the ASD group has difficulty with processing emotion and faces. These differential findings have implications for how one might consider targeting social skills remediation in the two groups. Finally, our findings contribute to the growing literature demonstrating the high degree of comorbidity among NDDs and perhaps the need to reconsider our diagnostic categories and labels.

## Data Availability Statement

The raw data supporting the conclusions of this article will be made available by the authors, without undue reservation.

## Ethics Statement

The studies involving human participants were reviewed and approved by the Hospital for Sick Children and Holland Bloorview Research Ethics Boards. Written informed consent to participate in this study was provided by the participants’ legal guardian/next of kin.

## Author Contributions

KS and MMV completed data analyses, drafted the manuscript, and contributed to interpretation and revisions. EWP contributed to drafting of the manuscript and interpretation of the data. KV contributed to recruitment and revisions. JC, RS, SG, RN, and MA contributed to the recruitment and confirmation of diagnoses of the NDD participants. AI contributed to management of the project. EK contributed to the recruitment and confirmation of diagnoses of the NDD participants and revised the manuscript. JPL contributed to project management, funding, and interpretation. EA contributed to the project management, funding, recruitment, and confirmation of diagnoses for the NDD participants and revision of the manuscript. MJT contributed to project management, funding, data interpretation, drafting, and revising the manuscript. All authors contributed to the article and approved the submitted version.

## Funding

Funding was provided for the POND data collection by the Ontario Brain Institute (IDS-I l-02) to EA and JPL, and by the Canadian Institutes of Health Research (CIHR) [MOP-119541; MOP-142379] to MJT.

## Conflict of Interest

EA has served as a consultant to Roche and Quadrant Therapeutics, has received consultation fees from Roche, holds a patent for the device, ‘Anxiety Meter’, and has received in kind support from AMO pharma, royalties from APPI and Springer, and editorial honoraria from Wiley.

The remaining authors declare that the research was conducted in the absence of any commercial or financial relationships that could be construed as a potential conflict of interest.

## Publisher’s Note

All claims expressed in this article are solely those of the authors and do not necessarily represent those of their affiliated organizations, or those of the publisher, the editors and the reviewers. Any product that may be evaluated in this article, or claim that may be made by its manufacturer, is not guaranteed or endorsed by the publisher.

## Footnotes

^1^ http://www.macbrain.org/resources.htm

^2^ http://www.neurobs.com

^3^ https://www.nitrc.org/projects/nbs
